# P42 Leptospira in the hotseat: a case from the ITU at York Teaching Hospital

**DOI:** 10.1093/jacamr/dlaf230.049

**Published:** 2025-12-04

**Authors:** Ammara Asif, Alice Tebboth, Natalia Turek, Katrina Blackmore

**Affiliations:** Department of Microbiology, York and Scarborough Teaching Hospitals NHS Foundation Trust; Department of Microbiology, York and Scarborough Teaching Hospitals NHS Foundation Trust; Department of Microbiology, York and Scarborough Teaching Hospitals NHS Foundation Trust; Department of Microbiology, York and Scarborough Teaching Hospitals NHS Foundation Trust

## Abstract

**Background:**

Leptospirosis is one of the most widespread zoonotic infections worldwide, though it remains relatively uncommon in the United Kingdom, with fewer than 100 cases reported annually. Humans are incidental hosts, typically acquiring the infection through contact with water or soil contaminated by the urine of infected animals, particularly rodents. Clinical manifestations vary widely, ranging from mild, self-limiting illness with fever, diarrhoea and myalgia to a fulminant, life-threatening form known as Weil’s disease, characterized by hepatic and renal dysfunction, pulmonary involvement and haemorrhagic complications.

**Case presentation:**

We report the case of a previously healthy Caucasian male in his forties who presented with a 3 day history of fever, vomiting and profuse watery diarrhoea. On arrival, investigations revealed acute kidney injury, thrombocytopenia and deranged liver function tests. CT imaging of the abdomen and pelvis was unremarkable, and peripheral blood film excluded haemolysis. Extensive microbiological testing, including hepatitis serology, HIV, CMV, EBV, Legionella, respiratory viral PCR and blood cultures, was initiated. Further history identified recent occupational exposure to contaminated water in a utility role, including contact with rat nests, as well as recent shellfish consumption. The patient’s condition rapidly deteriorated, with worsening renal function, jaundice, encephalopathy and acute respiratory distress syndrome (ARDS), necessitating intensive care admission and multiorgan support. Empirical antimicrobial therapy was commenced with a cephalosporin. Despite treatment, his respiratory and renal function declined, requiring intubation and continuous venovenous haemofiltration. On Day 3, leptospirosis was confirmed by reference laboratory testing (positive IgM and PCR), and therapy was refined to IV benzylpenicillin. The patient’s clinical status subsequently improved, allowing successful extubation and weaning off renal replacement therapy. He completed a 14 day antibiotic course and made a full recovery.

**Discussion:**

Icteric leptospirosis (Weil’s disease) develops in approximately 10% of symptomatic cases and can present with fever, jaundice, renal failure, ARDS, pulmonary haemorrhage, myocarditis, rhabdomyolysis and conjunctival suffusion. While mild disease is often self-limiting, severe cases demand prompt recognition, rapid initiation of appropriate IV antibiotics such as penicillin or ceftriaxone, and comprehensive supportive management. This case exemplifies severe leptospirosis acquired through occupational exposure, underlining the importance of early recognition, multidisciplinary care and public health awareness.

**Conclusions:**

This report reinforces the need for a low threshold of suspicion when evaluating patients with acute febrile illness and unexplained organ dysfunction, particularly in those with relevant occupational or environmental exposures. The protean manifestations of leptospirosis often delay diagnosis, with serious consequences if treatment is not initiated early. Clinicians must remain vigilant, even in regions where incidence is low, as travel, occupational risks and climate-related factors may increase case numbers. Heightened awareness among supported by timely diagnostic testing and coordinated multidisciplinary care, is essential to improve outcomes in this potentially fatal but treatable infection.

